# Development and implementation of a novel, mandatory competency-based medical education simulation program for pediatric emergency medicine faculty

**DOI:** 10.1186/s41077-021-00170-4

**Published:** 2021-05-06

**Authors:** Jonathan Pirie, Jabeen Fayyaz, Mireille Gharib, Laura Simone, Carrie Glanfield, Anna Kempinska

**Affiliations:** 1 Division of Emergency Medicine, The Hospital for Sick Children, 555 University Ave., Toronto, ON M5G 1X8 Canada; 2 PEM Simulation Program, Toronto, Canada; 3 University of Toronto, Toronto, Canada

**Keywords:** Simulation, Competency-based medical education, Procedures, Resuscitation, Continuing professional development

## Abstract

**Background:**

Maintaining acute care physician competence is critically important. Current maintenance of certification (MOC) programs has started to incorporate simulation-based education (SBE). However, competency expectations have not been defined. This article describes the development of a mandatory annual SBE, competency-based simulation program for technical and resuscitation skills for pediatric emergency medicine (PEM) physicians.

**Methods:**

The competency-based medical education (CBME) program was introduced in 2016. Procedural skill requirements were based on a needs assessment derived from Royal College PEM training guidelines. Resuscitation scenarios were modified versions of pre-existing in-situ mock codes or critical incident cases. All full-time faculty were required to participate annually in both sessions. Delivery of educational content included a flipped classroom website, deliberate practice, and stop-pause debriefing. All stations required competency checklists and global rating scales.

**Results:**

Between 2016 and 2018, 40 physicians and 48 registered nurses attended these courses. Overall course evaluations in 2018 were 4.92/5 and 4.93/5. Barriers to implementation include the need for many simulation education experts, time commitment, and clinical scheduling during course events.

**Conclusion:**

We have developed a mandatory simulation-based, technical, and resuscitation CBME program for PEM faculty. This simulation-based CBME program could be adapted to other acute care disciplines. Further research is required to determine if these skills are enhanced both in a simulated and real environment and if there is an impact on patient outcomes.

**Supplementary Information:**

The online version contains supplementary material available at 10.1186/s41077-021-00170-4.

## Background

Maintaining physician competence is critically important in acute care settings in order to deliver high-quality, evidence-based care. Current maintenance of certification (MOC) programs require mostly passive learning strategies. Simulation-based education (SBE), often in the form of in situ mock codes, has been widely adopted for post-graduate training. Efforts to incorporate simulation into MOC for practicing physicians have recently been introduced in some disciplines; however, performance is not linked to competency expectations [1, 2]. Annual requirements for competency in simulation-based procedural and resuscitation skills would ensure that physicians in acute care settings maintain their competency in critical lifesaving skills.

Physician knowledge decay is a well-known phenomenon after post-graduate training. In fact, skill decay has been demonstrated in numerous cardiopulmonary resuscitation (CPR)-based courses [3–8]. To maintain competence, emergency physicians need to participate in continuing medical education to ensure updated medical knowledge and skill acquisition especially for critical procedures [9]. The American Society of Anesthesiologists (ASA) introduced simulation-based education into MOC for Anesthesia in 2010 [1]. Upon completion of the simulation scenarios, participants are required to identify 3 areas for practice improvement and incorporate them into their future daily practice [2]. Participants receive MOCA credits after completion of the program; however, they are not required to pass the simulation cases in order to complete the process.

Some studies have shown that participant satisfaction is greater with simulation-based workshops and courses compared to traditional lecture-based courses [10–13]. Simulation has the advantage of being utilized not only as a training tool, but also as a framework to assess teamwork principles, leadership, and communication skills [14–17]. Skills developed during simulation training are transferable to patient care, resulting in significant improvements in patient outcomes [18–22]. Despite these benefits, Pirie et al. demonstrated that PEM physicians participating in weekly divisional interprofessional in situ simulations averaged only 1.25 sessions per year and team skills plateaued with time [23].

Competency-based medical education (CBME) has attracted the attention of educators and accreditation bodies [24–26] as it allows competency measurement for specific skills by being outcome-based and promotes learner-driven skills acquisition [25–27]. Frank et al. define CBME as “an approach to preparing physicians for practice that is fundamentally oriented to graduate outcome abilities and organized around competencies” [28]. CBME is currently being implemented primarily in post-graduate training programs but not with practicing clinicians.

Although it is well known that simulation-based education addresses many educational and competence assessment needs for physicians, its utilization in a competency format for faculty members in pediatric emergency medicine (PEM) has not to our knowledge been previously studied. This paper describes the development and implementation of a mandatory simulation based CBME program for faculty in PEM.

## Rationale

We developed a mandatory simulation competency-based procedural and resuscitation program in pediatric emergency medicine. In this report we describe the program, the curriculum from 2016 to 2018, and the applicability to other acute care settings aiming to adopt similar programs.

## Methods

This program was implemented in 2016 in the emergency department of a tertiary care pediatric hospital. At the time of implementation, there were 28–30 full-time staff MDs, 6–8 half-time or greater contract staff MDs, 16 PEM fellows, 6–7 advanced training fellows (e.g., simulation, POCUS), and over 100 RNs. In 2018, the ED had 80,555 patient visits of which the Canadian Triage and Acuity Scores (CTAS) were CTAS 1 (1.1%), CTAS 2 (19.7%), CTAS 3 (45.3%), CTAS 4 (27.1%), and CTAS 5 (5.8%).

### Course development

The CBME program for PEM faculty was introduced in 2016 and initially included training and assessment of both procedural and resuscitation skills. Although not done a priori, our curriculum development included the following stages of Kern's 6-step approach to curricular development [29].

1. Problem identification—skills gaps identified, frequency of individual in situ simulations insufficient, recurring morbidity cases
2. Needs assessment—Royal College Training Objectives
3. Goals and objectives—competency in core technical and resuscitations skills
4. Educational strategies—asynchronous website modules, annual simulation-based training, competency testing
5. Implementation—leadership support, simulation centre resources, PEM education and clinical expertise, scheduling
6. Evaluation—course evaluations, faculty feedback (future study), effect on in situ simulation performance (future study), mastery testing (future study)

### Procedures skills content

Our division undertook a physician skill needs assessment based on existing Royal College of Physicians and Surgeons of Canada Objectives of Training in the Subspecialty of Pediatric Emergency Medicine and found that many physicians had not performed or infrequently performed many critical procedural skills. The top 4 ranked procedural skills were chosen for the first course from this needs assessment (see Table 1).

**Table 1:** Procedural skills stations

Procedures				
2016	Intubation/Glidescope	Central venous access	Chest tube	Intraosseous
2017	Intubation/Glidescope	Cricothyroidotomy	Chest tube	Intraosseous
2018	Intubation/Glidescope	Cricothyroidotomy	Chest tube	Intraosseous

Equipment: Intubation—neonatal, infant, and child torso (Laerdal, USA); Life/Form cricothyroidotomy simulator (Nasco); central venous access child (SimuLab); chest tube (made locally with pork ribs and multi-layer silicone); IO (EZ-IO drill kit and legs)

The pre-existing ISMC committee which consisted of 5 PEM simulation education faculty, 1 interprofessional education nursing specialist, 1 clinical support nurse, and 1 respiratory therapy education specialist were responsible for case selection and development.

### Resuscitation case content

Resuscitation scenarios were developed based on pre-existing in situ mock code (ISMC) cases which incorporated both Pediatric Advance Life Support (PALS) algorithms as well as cases which challenge participants’ team or crisis resource management (CRM) skills (see Table 2). Resuscitation station content was initially derived primarily by the primary author (JP) and members of the PEM in situ team training committee. It was decided that one case per session would include a PALS algorithm sequence. A second case was selected based on the need to order multiple medications, testing leaders’ ability to prioritize medication orders and the team’s ability to deliver the medications in a timely manner using excellent closed-loop-communication, as this was identified as the most common skills gap in our in situ team training program [23]. Finally, the third case was arbitrarily decided based on either new updated guidelines (sepsis, trauma—massive hemorrhage) or potential but rare cases (sedation with laryngospasm). Changes to the program were decided by the PEM simulation committee based on feedback from evaluations as well as morbidity case reviews.

**Table 2:** Resuscitation skills stations

Resuscitation				
2016	CPR/ventilation and defibrillation	Myocarditis + VT	CAH with hyperkalemia	Sepsis
2017	CPR/ventilation and defibrillation	Myocarditis + VT	CAH with hyperkalemia	Sepsis
2018	CPR/ventilation and defibrillation	Unstable SVT	Sedation—laryngospasm	Trauma—GSW massive hemorrhage

Equipment: Sim NewB, SimBaby, Sim Junior (Laerdal, USA)

*CPR* Cardio-pulmonary resuscitation, *VT* Ventricular fibrillations, *SVT* Supraventricular tachycardia, *CAH* Congenital adrenal hyperplasia, *GSW* Gunshot wound

Because the CBME program was developed as an adjunct to the existing ISMC team training program, Institutional Ethics Review was not required.

### Course delivery

The CBME program initially consisted of 2 half-day courses of procedures and 2 half-day courses of resuscitation per year. For ease of administration the half-days were combined into 2 full-day courses after the first year. Each MD faculty is required to complete one procedural and one resuscitation simulation course per year. In 2018, point of care ultrasound (POCUS) was added to the procedural half-day component of the program. Nurses traditionally are expected to have a full day of education per year and so those assigned on the CBME day were active participants.

PEM RNs completed a RN-focused procedural skills education half-day separately from the MD participants and then joined the half-day resuscitation team-based competency portion of the course. Due to RN staffing shortages, a maximum of 8 RNs were permitted to attend any given CBME session, resulting in 2 RNs per group. RNs not able to attend the CBME course were scheduled into the monthly interprofessional in situ mock trauma simulations. During non-CBME months, the nurses participated in the existing in situ mock traumas.

Each resuscitation group consisted of 2–4 staff physicians and 2 RN participants which enhanced the interprofessional teamwork of the sessions. Participants were all expected to play a role which they would normally do in a real scenario. A debriefing session was held following each resuscitation scenario with the intention of clarifying medical issues arising in the case and discussing crisis resource management aspects including interprofessional teamwork.

The range of MD participants per course was 10–19 and the number of instructors ranged from a minimum of 8 for a half-day and 16 for a full-day course, averaging approximately 1 instructor per POCUS/technical and 2 instructors per resuscitation stations. In total, 40 PEM physicians and 48 PEM nurses participated in the program from 2016 to 2018. The mean percentage of MDs participating per year was 85.4% and the mean percentage instructing per year was 42.8%.

### Website content

An asynchronous flipped classroom approach was utilized. The RN-specific procedures eLearning was available on the SickKids ED intranet education page. A separate website with MD specific procedures and interprofessional (MD and RN) resuscitation case modules was created. Each learning module consisting of online videos and content-specific reading material was made available for the participants to review prior to the course. Website material was prepared by PEM and simulation experts as well as our interprofessional nurse education specialist (CG) based on RCPSC core knowledge requirements for PEM trained physicians as well as divisional clinical pathways, order sets, and procedural guidelines. Website material was password protected for participants. The competency checklists for each station were also available on the website (discussed below) so that participants may familiarize themselves with them beforehand. All MD and RN participants were expected to review the content material prior to taking the course. Station content included the following:

1. Station objectives
2. Video instruction
3. Reading material: e.g., guidelines, journal articles, textbook chapters
4. Checklist
5. Additional resources or links

### Competency testing

Both Checklists and Global Rating Scale (GRS) specifically designed for each individual procedure or resuscitation station were used in order to assess competency throughout the full-day course. Checklists were designed separately for each station; some were modified from previously validated Objective Score of Technical Skills (OSAT) [30], while others were designed by PEM faculty and PEM educational experts with expertise in those skills (procedural) or content area (resuscitation) (see Additional file 1 MD for an example of a procedural checklist). Nursing used locally derived checklists for procedural skills (see Additional file 1 RN for an example of a procedural checklist).

For resuscitation scenarios, checklists included Crisis Resource Management components in order to highlight the importance of team functioning during resuscitations. While checklists listed every step in performing a procedural skill or accurately running a resuscitation scenario, the most important of these steps were highlighted in bold. Participants were required to achieve all bolded checklist items in order to achieve overall competence on the GRS. Several studies have assessed validity of GRS in the emergency setting [31, 32] and a systematic review has demonstrated some of the advantages of GRS over checklists [33]. Therefore, the decision was made to use checklists formatively, with the most important steps highlighted in bold. The GRS was used summatively to determine competence (see Additional file 2). Participants were required to achieve all checklist items in bold as a minimum passing standard (MPS) to achieve overall competence on the GRS.

Instructors with expertise within PEM education were identified and recruited to teach and evaluate each station. Instructors were directed on the components of the checklists and GRS, and asked to familiarize themselves with the website course material. Guidance on using the checklists and GRS to assess for competency was also given. A core group of instructors was identified as the course progressed, although instructors needed to rotate through competency days themselves as participants.

For procedural competence testing, all participants utilized repeated deliberate practice, an education methodology of repeated skills and resuscitation training with feedback, and then completed a final competency testing [34–37]. Unsuccessful participants were asked to repeat the testing until competency was met. Failure to meet competency by the end of the course resulted in a failure to pass the station.

For resuscitation competence testing, stop-pause debriefing [38] was utilized to reinforce learning and key scenario competencies followed by a complete scenario for GRS competency. Competency was defined a priori as team competence rather than individual competence, as the performance of the team ultimately determines outcomes in real-life cases (see Additional files 3 and 4 for examples of resuscitation station checklists and GRS). Unsuccessful team performance would result in teams needing to repeat the scenario until competency was achieved.

Although individuals and teams infrequently were unsuccessful, performance data will be analyzed in a separate study.

No formal rater training was utilized for the checklist and global rating scores. The majority of raters had used the checklists for other courses and our in situ mock code program so consistency of scoring was likely very high.

### Cost

Cost estimates were approximated (see Table 3) and include (1) faculty time—both teachers and learners; (2) equipment including models for procedures; (3) room rental (covered by institutional simulation program); and (4) supplies.

**Table 3:** Average financing for the CBME program (projected and actual $CAN) per year

Course content	Items	Projected cost	Actual cost
**Procedures** (2 half-day sessions per year)	*Simulation space* • including simulation specialist	$1750	No cost
	*Human resources/session* • Nursing instructors: 1 × 4 h at $40/h per session = $160/session • MD faculty: no cost	$320	No cost
	*Supplies* • $200 per session	$400	$400
**Resuscitation** (2 half-day sessions per year)	*Simulation space* • including simulation specialist	$1750	No cost
	*Human resources* • Nursing instructors: 4 × 4 h at $40/h per session = $640/session • MD faculty: no cost	$1280	No cost
	*Supplies* 1. $50	$100	$100
**Total cost**		$5600	$500

### Course evaluations

Evaluations of both instructors and course were initiated with program implementation. From 2016 to 2018, the average instructor evaluation for POCUS 4.86 (range 4.63–5.0), procedural was 4.95 (range 4.5–5.0), and resuscitation was 4.81 (range 4.63–5.0). Overall course scores started in 2018 and mean scores were 4.92 and 4.93 for each course.

Overall comments for the course were very favorable. Technical skills comments included “great stations,” “friendly and positive learning environment,” “enjoyed viewing uncommon but potential complications to common procedures in the ED,” and “deliberate practice awesome”. Comments from resuscitation stations included “great for nursing to participate,” “hands on and interactive with constructive feedback in real time and conductive to my learning during scenario,” “makes people feel good even when feedback is constructive/negative”, “never felt judged or criticized”, and “love that it was a group scenario and focus was on team and communication.” Some barriers were also identified: “more facilitators to speed up assessments,” “long day, resuscitation sessions shorter,” “more nurses per group.”

## Discussion

The authors report the development, implementation, and participant evaluations of an innovative multimodal continuing education course for faculty competency maintenance and assessment. Online learning material included key articles, clinical guidelines, videos, checklists, and online self-assessment tools. The hands-on procedures incorporated deliberate practice and resuscitations were debriefed using stop-pause methodology. A synthesis of systematic reviews showed that CME activities that were more interactive, used more methods, and involved multiple exposures were more likely to lead to improved physician performance and patient outcomes [39].

### Challenges

Scheduling of both participants and instructors was and continues to be a challenge. Given the need to cover the clinical workload on CBME course days and individual’s academic responsibilities, developing a balanced schedule was difficult. On average 1–3 staff would “drop out” in the week leading up to the course. As these sessions are mandatory, most of these individuals would then request to participate in the following session leading to larger group sizes which ultimately impacted flow, timing, and instructor scheduling. The number of MD participants ranged from 9 to 17 per session. Additionally, the division continues to add new staff as the clinical and academic load has increased significantly each year.

The number of instructors who have either simulation expertise or technical expertise could be a challenge for smaller programs. There are five staff with simulation fellowship training or equivalent as well as numerous faculty who participate in the simulation instruction of post-graduate trainees from junior resident through to PEM fellows, most of which have taken a simulation instructor workshop. Additionally, many staff have clinical expertise which was utilized for either technical or scenario case development and instruction. Despite this broad education expertise, approximately 12–15 MD staff educators and 4–5 RN educators are required per session, meaning that many of the simulation “experts” were required to teach multiple course in a row. As a result, these faculty have not been able to take the course as a participant on annual basis, as mandated by the program. For smaller programs with fewer simulation educators, it may be difficult to run a program of this size.

Nursing participation was more challenging for the physicians for several reasons. Firstly, there are over 100 nurses in our division and so by sheer numbers it would very difficult to complete the CBME course in a given year. Additionally, the funding model for nurses only permits a limited number of paid education days per year. This allowed for just less than one-third of the RN group per year to participate. The remainder of the nurses continued to participate in the in situ program plus the pre-existing procedural training annually.

### Next steps

Station and content development are important components of the program. Subsequent changes were iteratively made based on feedback from faculty evaluations and simulation/resuscitation expert panel. Ultimately, it will be important to define a set curriculum which can be rotated over subsequent years which represent both common and infrequent but high-risk critical skills. Potential solutions include repeating of a needs assessment as well as continuing to utilize quality reviews as a source for new case development.

Although procedural and POCUS skills were easily evaluated individually, the resuscitation stations were evaluated based on team competence. Ideally, our competency evaluations should also include leader competency. Although most physicians lead at least one case, several courses with larger participant numbers hindered all MDs from participating in the lead position. Also, competence in one case is not necessarily generalizable to other station content. Individually testing all MDs across all cases would require repeating the cases 3 or 4 more times for each group which is not feasible in a half-day format. In the future, we hope to review the completion rates and incorporate strategies to move from a competency model to a true mastery model.

Future research is required in order to evaluate the impact of this innovative program. Following Kirkpatrick’s hierarchy, evaluation of education programs happens at four levels: reactions, learning (knowledge, skills, attitudes), behavior (simulated or clinical), results (patient outcomes) [40]. As this is a new program, evaluation across all four levels is recommended. Currently, we are evaluating our program at the first three levels and hope to report on these findings in the near future. Additionally, feasibility is an extremely important consideration, and the ability of other acute care disciplines to adopt this program will depend on resources, finances, and leadership buy-in.

## Conclusion

We have developed an annual mandatory simulation-based technical, POCUS, and resuscitation CBME program for PEM faculty. Although challenges around scheduling exist, the course was extremely well received by participants with excellent participation rates. Ensuring lifelong competence in acute care skills is essential for PEM physicians and nurses. This program addresses gaps in the traditional models of MOC and skills decay associate with life support courses. Our simulation-based CBME program could be adapted and generalized to other acute care disciplines. Further research is required to determine if these skills are enhanced both in a simulated and real environment and if there is an impact on patient outcomes.

## Supplementary Information

**Additional file 1.** MD: Procedural Checklist. RN: Procedural Checklist.

**Additional file 2.** Procedural Global Rating Scale (GRS).

**Additional file 3.** Resuscitation Checklist.

**Additional file 4.** Resuscitation Global Rating Scale (GRS).

## Data Availability

The datasets used and or analyzed during the current study are available from the corresponding author unreasonable request.
