# Supplementary material for: Development and implementation of a novel, mandatory competency-based medical education simulation program for pediatric emergency medicine faculty
Source: Adv Simul (Lond). 2021 May 6;6:17. doi: 10.1186/s41077-021-00170-4 (PMC8101101; doi:10.1186/s41077-021-00170-4)
Supplement: Supplementary file 1 — Additional file 1. MD: Procedural Checklist. RN: Procedural Checklist. [file 41077_2021_170_MOESM1_ESM.zip › APPENDIX A Procedural Checklist.docx]

Additional file 1: MD: Procedural Checklist

Needle Cricothyrotomy Checklist

Pediatric Emergency Medicine

Competency-based training evaluation

Staff being evaluated: ________________________________

Instructor: _________________________________________

Date: ______________________________________________

| Step | Skill | Done incorrectly / not done | Done correctly |
| --- | --- | --- | --- |
| 1 | Verbalizes indications , contraindications and complications of performing cricothyrotomy |  |  |
| 2 | Done PPE ( as appropriate ) and gloves |  |  |
| 3 | Identify and set up equipment needed |  |  |
| 4 | Applies sterile gown and appropriately drapes patient |  |  |
| 5 | Verbalize need for anesthetic ( if necessary ) |  |  |
| **6** | **Identify the cricothyroid membrane between the cricoid and thyroid cartilage** |  |  |
| **7** | **Palpate the cricothyroid membrane and stabilizing it make a vertical incision in the midline** |  |  |
| **8** | **Make a small puncture in the skin using the 18G needle over the center of cricothyroid membrane** |  |  |
| **9** | **Insert the IV cannula at an angle of 45 degree downward through the cricothyroid membrane until a pop is felt** |  |  |
| **10** | **Attach the syringe and confirm the insertion via the immediate return of air in the syringe** |  |  |
| **11** | **Attach the catheter needle hub to a 3 mm endotracheal tube adaptor (or 7.5mm ETT adaptor with 3 ml syringe)** |  |  |
| 12 | Connect the adaptor to oxygen tubing via a Y-connector or flow modulator |  |  |
| **13** | **Set the oxygen flowmeter at 15 L per minute – ventilate with I:E ratio greater than 1:4 seconds** |  |  |
| 14 | Manually guard the plastic cannula to prevent it from kinking and prepare for more definitive airway |  |  |
