# Supplementary material for: Development and implementation of a novel, mandatory competency-based medical education simulation program for pediatric emergency medicine faculty
Source: Adv Simul (Lond). 2021 May 6;6:17. doi: 10.1186/s41077-021-00170-4 (PMC8101101; doi:10.1186/s41077-021-00170-4)
Supplement: Supplementary file 1 — Additional file 1. MD: Procedural Checklist. RN: Procedural Checklist. [file 41077_2021_170_MOESM1_ESM.zip › Appendix A RN - EZIO FACILITATOR GUIDE.docx]

Additional file 1: RN: Procedural Checklist

**RN EZIO FACILITATOR GUIDE**

**Facilitator set-up:** Have the following equipment available; EZIO needles pre-inserted in mannequin leg or IV pad, 10cc leur lock syringe, stabilizers, 3 sizes of needle.

| **Question** | **Key Points for Competency Check** |
| --- | --- |
| What is the indication for IO insertion? | - Any time vascular access is difficult to obtain in critical situation |
| Name 2 contraindications to IO insertion. | - Fracture of target bone - Osteogenesis imperfecta (or similar bone disease) - Previous orthopedic procedure at insertion site - IO in target bone in past 48 hours - Infection at area of insertion - Excessive tissue or absence of adequate anatomical landmarks |
| Where is the EZIO equipment stocked?  And which size needles do we stock? | - Yellow zipper bag IO kit in Resus/Trauma room, Stepdown room, code blue cart - Back up supplies in storage room behind East station - Pink (15 mm, 15 G, infant) - Blue (25 mm, 15 G, child) - Yellow (45 mm, 15 G large adult)   Note: choice of needle size is not simply the weight on the package. Also consider anatomy and tissue depth at site. Black 5mm mark must be visible above skin when needle set touching bone prior to drilling. |
| What do we need to monitor for when an IO is infusing? | - Signs of infiltration - Signs of compartment syndrome |
| Demonstrate securing the site using a stabilizer. | - Place stabilizer over the hub - Attach a connection set to the hub (primed if not drawing bloodwork) - Secure by twisting clock-wise - Pull tabs off adhesive - Apply adhesive to skin - Aspirate blood bone marrow & flush with NS |
| What labs can be run with bone marrow?   - What if there is no blood return – does it mean it is not in? | - All standard resus/trauma bloodwork, as long as the order specifies IO so the lab knows the source of the sample for reference ranges   Note: The iSTAT is calibrated for venous, arterial, and capillary samples only. |
| What medications can be infused? | - All standard resus/trauma medications |
| How do you remove IO? | - Remove extension set and dressing - Stabilize hub & attach a leur lock syringe - Quarter twist clockwise and pull straight up (don’t rock – could bend catheter) |
