# Supplementary material for: Development and implementation of a novel, mandatory competency-based medical education simulation program for pediatric emergency medicine faculty
Source: Adv Simul (Lond). 2021 May 6;6:17. doi: 10.1186/s41077-021-00170-4 (PMC8101101; doi:10.1186/s41077-021-00170-4)
Supplement: Supplementary file 2 — Additional file 2. Procedural Global Rating Scale (GRS). [file 41077_2021_170_MOESM2_ESM.docx]

Additional file 2: Procedural Global Rating Scale (GRS)

**Global Rating Scale for Procedural Skills**

**Staff being evaluated:**

**Procedure:**

**Circle the applicable number on the scale for each skill listed below:

| Flow and Efficiency | 1  Frequently stopped procedure and/or made many unnecessary moves | 2 | 3  Efficient progression of procedure but made some unnecessary moves | 4 | 5  Effortless flow of procedure and maximum efficiency |
| --- | --- | --- | --- | --- | --- |
| Respect for tissue | 1  Frequently used unnecessary force on tissue or caused damage | 2 | 3  Careful handling of tissue but occasionally caused inadvertent damage | 4 | 5  Consistently handled tissues appropriately with minimal damage |
| Instrument handling | 1  Repeatedly makes tentative or awkward moves with instruments | 2 | 3  Competent use of instruments but occasionally appeared stiff or awkward | 4 | 5  Fluid moves with instruments and no awkwardness |
| Overall performance | 1  Very Poor | 2 | 3  Competent | 4 | 5  Mastery |

Comments: ____________________________________________________________________________________________________________________________________________________________

______________________________________________________________________________

______________________________________________________________________________
