# Supplementary material for: Development and implementation of a novel, mandatory competency-based medical education simulation program for pediatric emergency medicine faculty
Source: Adv Simul (Lond). 2021 May 6;6:17. doi: 10.1186/s41077-021-00170-4 (PMC8101101; doi:10.1186/s41077-021-00170-4)
Supplement: Supplementary file 3 — Additional file 3. Resuscitation Checklist. [file 41077_2021_170_MOESM3_ESM.docx]

Additional file 3: Resuscitation Checklist

**MYOCARDITIS/UNSTABLE VT CHECKLIST**

| Action |  | Not Done | Done Poorly or Partially | Done Well | Team Function |
| --- | --- | --- | --- | --- | --- |
| Oxygen mask |  |  |  |  |  |
| Monitor |  |  |  |  |  |
| IV Access |  |  |  |  |  |
| **Blood work:** | Accu-check, iSTAT, & shock bloodwork |  |  |  |  |
| **Fluids: NS** | 1^st^ (20 cc/kg) |  |  |  |  |
|  | 2^nd^ (20 cc/kg) |  |  |  |  |
| Recognize VT | Establish that unstable/pulseless |  |  |  | Time to recognize |
| **CPR** | QCPR (rate 100-120), depth > 1/3 AP, full recoil |  |  |  |  |
|  | CPR:Ventilation 15:2 (proper call out) |  |  |  |  |
|  | 2 minute cycles – no interruptions > 10 sec |  |  |  |  |
| **Drugs** | Epinephrine 1:10,000 0.1 ml/kg (0.01 mg/kg) q3-5 minutes |  |  |  |  |
| **Defibrillation** | Appropriate PAD size and location (child size, location depending on mannequin size either front or front and back) |  |  |  |  |
|  | Appropriate amount 2-4-4 j/kg |  |  |  |  |
| **RSI** | AMPLE (especially allergies)  Equipment: SCOPES |  |  |  |  |
| **Post Intubation Care** | Calorimetric CO2, CXR or POCUS, ETCO2, etc. |  |  |  |  |
|  | 1 breath q 6-8 sec (8-10 breaths/min) |  |  |  |  |
| **Drugs** | Amiodarone or lidocaine |  |  |  |  |
| **Consultants** | ICU  Travel modality |  |  |  |  |
| **Situational Awareness** | Recognizes rhythm change (as above) *Perception* |  |  |  |  |
|  | Verbalizes Mental Model (what’s going on – possible myocarditis) *Comprehension* |  |  |  |  |
|  | Anticipates Next actions. *Projection* |  |  |  |  |
| **Resuscitation Order Sheet** | Used appropriately – documentation by team member |  |  |  |  |
