# Supplementary material for: Development and implementation of a novel, mandatory competency-based medical education simulation program for pediatric emergency medicine faculty
Source: Adv Simul (Lond). 2021 May 6;6:17. doi: 10.1186/s41077-021-00170-4 (PMC8101101; doi:10.1186/s41077-021-00170-4)
Supplement: Supplementary file 4 — Additional file 4. Resuscitation Global Rating Scale (GRS). [file 41077_2021_170_MOESM4_ESM.docx]

Additional file 4: Resuscitation Global Rating Scale (GRS)

**Global Rating Scale for Resuscitation Skills**

**Staff (or Team) being evaluated:**

**Skills being assessed: (circle appropriate station)**

**Defib CPR PALS/Situational Awareness CAH/Comm Shock/Leadership & Roles**

**Circle the applicable number on the scale listed below:

| Overall performance | 1  Very Poor | 2 | 3  Competent | 4 | 5  Clearly Superior |
| --- | --- | --- | --- | --- | --- |

Comments: ______________________________________________________________________________

______________________________________________________________________________

______________________________________________________________________________

______________________________________________________________________________
